# Assessment of suitable region of *Asparagus cochinchinensis* (Lour.) Merr. under different climatic conditions in China by the MaxEnt model and HPLC analysis

**DOI:** 10.1002/ece3.70354

**Published:** 2024-10-03

**Authors:** Tong Zhang, Xiangyang Lv, Qian Zhao, Caijuan Zhang, Honglin Yin, Shuyu Deng, Gui Yan, Guangzhi Wang, Xiaoyan Cao, Hong Ou, Gang Shen

**Affiliations:** ^1^ Chengdu Institute of Chinese Herbal Medicine Chengdu China; ^2^ Neijiang Academy of Agricultural Sciences Neijiang China; ^3^ Key Laboratory of the Ministry of Education for Medicinal Resources and Natural Pharmaceutical Chemistry, National Engineering Laboratory for Resource Development of Endangered Crude Drugs in Northwest of China, College of Life Science Shaanxi Normal University Xi'an China; ^4^ State Key Laboratory of Southwestern Chinese Medicine Resources Chengdu University of Traditional Chinese Medicine Chengdu China; ^5^ Hospital of Chengdu University of Traditional Chinese Medicine Chengdu China

**Keywords:** *Asparagus cochinchinensis* (Lour.) Merr., climatic factor, correlation, MaxEnt model, secondary metabolite

## Abstract

*Asparagus cochinchinensis* is a member of the Asparagaceae family whose medicinal part is the dried root tuber. The distribution of *A. cochinchinensis* and its secondary metabolites are closely associated with environmental factors, such as climate and soil properties. By establishing and optimizing a maximum entropy model, we analyzed and predicted the distribution pattern and migration direction of suitable habitats for *A. cochinchinensis* and determined the main environmental factors affecting the accumulation of secondary metabolites. Under current climatic conditions, the area of suitable habitats for *A. cochinchinensis* (208.38 × 10^4^ km^2^) accounts for 21.71% of the land area of China, and the areas of lowly, moderately, and highly suitable areas were 64.15 × 10^4^ km^2^, 113.66 × 10^4^ km^2^, and 30.57 × 10^4^ km^2^, respectively. Under future climate scenarios, the total area of suitable habitats hardly changes. The area of highly suitable habitats significantly decreases under the SSP1‐RCP2.6 scenario (to 83.22% of the current value) and the SSP3‐RCP7 scenario (to 48.94% of the current value), but eventually increases to 112.86% of the current value under the SSP5‐RCP8.5 scenario, which indicates that *A. cochinchinensis* might adapt better to a high‐carbon‐emissions scenario. Under different climate scenarios, low‐impact areas mainly occur in southern China and will correspond 92.07% of the current suitable area. Highly suitable habitats primarily occur in the southeastern Sichuan Basin, northern Guangxi, eastern Guizhou, and western Hunan. HPLC analysis showed that the content of protodioscin (0.373%) and protogracillin (0.044%) in S2 was the highest. The total saponins contents of S1 and S2 were the highest, which were 35.6586 and 33.1262 mg/g, respectively. The total polysaccharide content of S9 was the highest (16.9467%). The total contents of saponins and polysaccharides in *A. cochinchinensis* were significantly, but oppositely, correlated with temperature, precipitation, and other factors. This study has identified environmental factors affecting the growth and quality of *A. cochinchinensis*, which has guiding significance for resource conservation and site selection for large‐scale cultivation.

## Graphical abstract

Species distribution model is used to predict the distribution of suitable habitats of *Asparagus cochinchinensis* (Lour.) Merr. in China. Precipitation and temperature are the key factors affecting the accumulation of active components in *A. cochinchinensis*. Saponins and total polysaccharides contents show different response patterns to climatic factors.

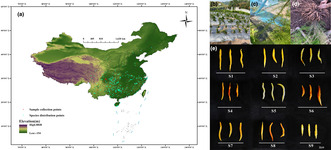

## 1 INTRODUCTION

Medicinal plants are biological resources necessary for producing active ingredients of medicines, and their growth and distribution are affected by various environmental factors, such as temperature, humidity, and soil properties (Cheng et al., 2018; Shen et al., 2024). Different medicinal plants exhibit distinct preferences for climatic factors. Regarding drought stress, moderate drought stress could promote the accumulation of secondary metabolites in *Scutellaria baicalensis* (Cheng et al., 2018). In contrast, in *Sophora alopecuroides* L. the biosynthesis of alkaloids could only be promoted under severe drought stress (Huang et al., 2024), and this plant is thus suitable for cultivation in arid areas. However, mint and *Pinellia ternata* are ideal for growing in hot and humid areas because they contain higher levels of secondary metabolites under humid conditions (Kumar et al., 2024; Wu et al., 2024). Therefore, to effectively use and develop medicinal plants, it is necessary to rationally and accurately identify suitable areas for their growth.

*Asparagus cochinchinensis* (Lour.) Merr. is one of the most widely known medicinal plants in the Asparagaceae family, and its medicinal part is the dried root tuber. In China, the areas where it is cultivated are mainly located in Sichuan, Guangxi, and Guizhou provinces. Modern pharmacology has found that *A. cochinchinensis* has antioxidant (Wang et al., 2024), anti‐inflammatory (Lee et al., 2009), immunostimulatory (Xie et al., 2024), antitumor (Zhang et al., 2023), and antidepressant effects (Jalsrai et al., 2016). The chemical constituents of *A. cochinchinensis* include saponins, polysaccharides, amino acids, lignans, and flavonoids, of which saponins and polysaccharides are the main active substances (Wang et al., 2022). As the principal drug in Erdong Decoction, *A. cochinchinensis* is often used for the intial treatment of diabetes (Yuan et al., 2018) and lung cancer (Zhao et al., 2012). Moreover, as *A. cochinchinensis* is a novel edible homologous traditional Chinese medicine, in China the demand for *A. cochinchinensis* is expected to increase in the future.

An ecological niche model, also known as a species distribution model, is a method of simulating the spatial distribution of species, predicting the potential responses of organisms to climate change, and determining the ecological niches of species on the basis of environmental variables (Brown et al., 2017; Brown & Anderson, 2014; Conolly et al., 2012). Such models have been employed worldwide by researches in disciplines such as bioecology and biogeography for analyzing the ecological conditions under which species occur and for predicting suitable habitats in different locations (Araújo & Guisan, 2006). By research and comparison of various existing ecological niche models, it has been found that the maximum entropy (MaxEnt) model has better predictive ability, and this is currently one of the most widely used models (Qin et al., 2017; Yang et al., 2013; Zhang et al., 2019). Thus far, the MaxEnt model has been employed to predict changing trends in potential habitats for many plant species, especially endangered species, such as *Homonoia riparia* Lour. (Yi et al., 2016), *Lebrunia busbaie* Staner (Imani Wa Rusaati & Won Kang, 2024), and *Taxus wallichiana* (Yousaf et al., 2022).

High‐performance liquid chromatography (HPLC) has the advantages of high sensitivity and accuracy and can effectively determine differences in the contents of secondary metabolites in medicinal plants from different regions (Gao et al., 2024; Ren et al., 2021; Shuai et al., 2022). Among the secondary metabolites of *A. cochinchinensis*, steroidal saponins are the main active ingredients, of which protodioscin and protogracellin are the main components. Moreover, protogracellin is the indicator compound for evaluation of the quality of medicinal materials from *A. cochinchinensis*, as specified in the Hong Kong Chinese Materia Medica Standards. A study has compared the contents of effective ingredients of *A. cochinchinensis* from different regions, and the results showed that *A. cochinchinensis* from Sichuan, Guizhou, and Yunnan was superior to that from Guangxi and Chongqing (Zhang et al., 2024). However, it has also been found that, from the perspective of the saponin content, *A. cochinchinensis* produced in Guangxi is of better quality than that produced in other areas (Xiong et al., 2011). Therefore, in this study the MaxEnt model and HPLC were utilized to analyze the influences of different environmental variables on the distribution of *A. cochinchinensis* from appropriate field data and network sampling data for this species and thus provide a scientific basis for the delineation of suitable areas for the planting and sustainable supply of *A. cochinchinensis*.

For the first time, we combined MaxEnt model with HPLC analysis and applied it to the distribution of suitable areas of *A. cochinchinensis*, providing an overall consideration of how environmental variables affect the accumulation of active ingredients. The purpose of this study was to: (1) study the main environmental variables that affect the distribution of *A. cochinchinensis*; (2) identify and infer suitable habitats for *A. cochinchinensis* under past, present, and future climate scenarios; (3) analyze the effects of habitat suitability on the contents of functional components in *A. cochinchinensis*; and (4) comprehensively evaluate the suitability of areas for cultivating *A. cochinchinensis*. The results of this study can provide a reference for land resource management and sustainable cultivation of *A. cochinchinensis*.

## 2 MATERIALS AND METHODS

### 2.1 Research area and data sources

**FIGURE 1:**
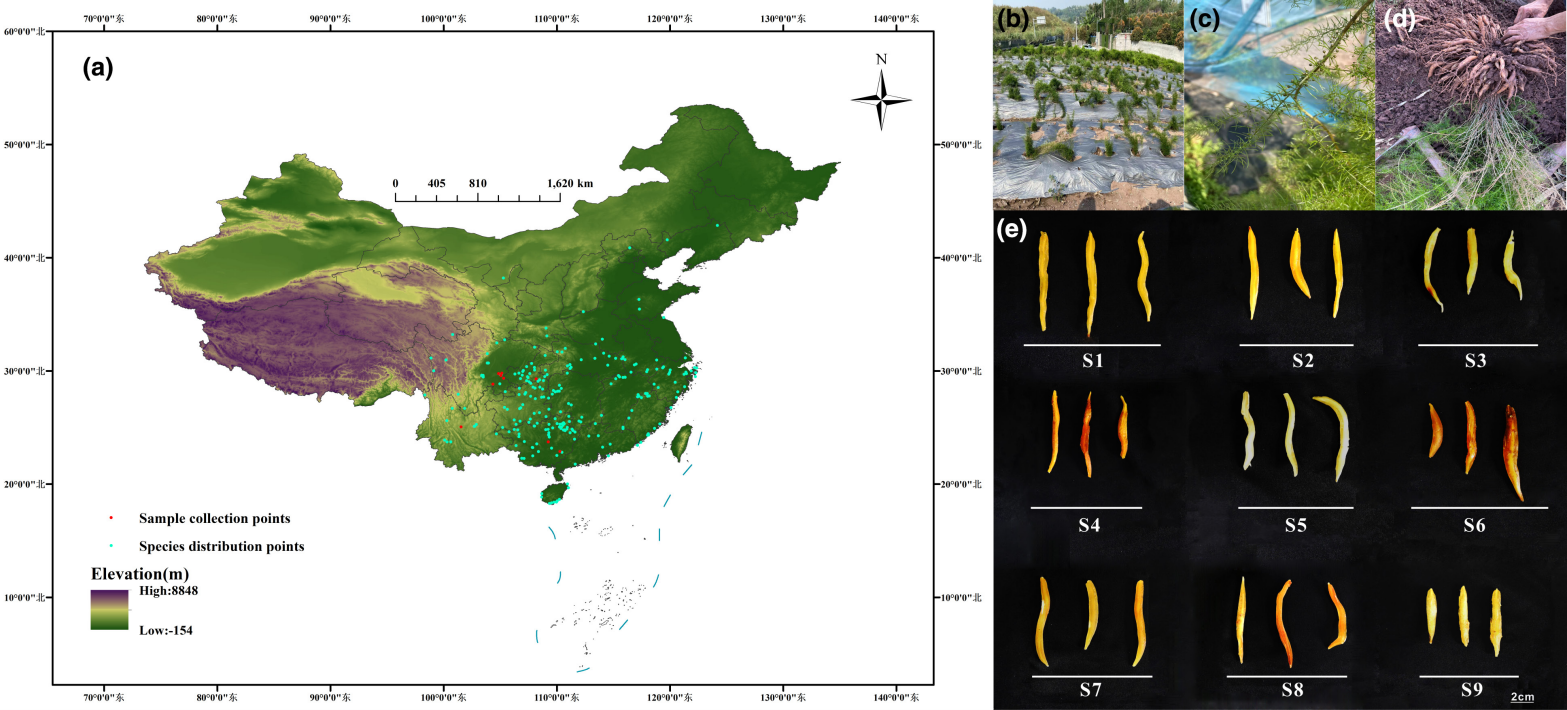
(a) Samples and species distribution information. (b–d) *Asparagus cochinchinensis* in the field. From Dongxing District, Neijiang City, Sichuan Province, China (105°4′28″ E, 29°35′36″ N), photograph by Tong Zhang. (e) Samples used in this study.

The research area covered the whole of China, with geographic coordinates ranging from 73.1° E to 135.5° E and from 17.7° N to 53.9° N. Distribution records for *A. cochinchinensis* were retrieved from public databases, including the Chinese Virtual Herbarium (https://www.cvh.ac.cn/), National Specimen Information Infrastructure (http://www.nsii.org.cn), and China National Knowledge Infrastructure (https://www.cnki.net/). According to previous research (Ye et al., 2020), the collected data were filtered, and non‐natural records were removed. Redundant data were deleted, and 259 valid samples were ultimately obtained, including a total of nine samples of *A. cochinchinensis* from Sichuan (5), Guangxi (2), Yunnan (1), and Chongqing (1). Figure 1 and Table S1 show information on the samples and species distribution sites.

The raw environmental data used in this study were downloaded from online databases including CHELSA (https://chelsa‐climate.org/), EarthEnv (http://www.earthenv.org/topography/), and ORNL DAAC (https://doi.org/10.3334/ORNLDAAC/1304), which contain data for recent (1981–2010) and future (2041–2070 and 2071–2100) eras. Forty‐seven environmental variables were used for the model predictions, and the spatial resolution of all the environmental data was 2.5 arc minutes (Table S2).

Considering the effects of the selected climate scenarios on the predictive model, we selected combinations of three SSPs and RCPs that correspond to five models of the atmospheric circulation (GFDL‐ESM4, IPSL‐CM6A‐LR, MPI‐ESM1.2‐HR, MRI‐ESM2.0, and UKESM1.0‐LL), namely, SSP1‐RCP2.6, SSP3‐RCP7, and SSP5‐RCP8.5. The severity of climate change under the three climate scenarios increases from SSP1‐RCP2.6 to SSP5‐RCP8.5. The predicted result layers corresponding to each SSP were arithmetically averaged to reduce the uncertainty that a single model of the atmospheric circulation might introduce into the distribution of potential habitats for species. Therefore, 31 climate datasets were used in this study, including one set of current data and 30 sets of future data. On the basis of the data for species distribution sites, sample collection sites, and the abovementioned 47 environmental variables, we first used the jackknife method to determine the importance of each variable to the model (Shcheglovitova & Anderson, 2013). Secondly, the Pearson's correlation coefficients between the 47 bioclimatic factors were calculated, and |*R*| ≥ .8 was used as the threshold to determine the significance of correlations between climatic factors. Finally, from each pair of significantly correlated variables, only the factor with the more substantial contribution was retained (Wei et al., 2021).

### 2.2 Model construction, optimization, and reliability testing

MaxEnt v3.4.4 software was used to construct a MaxEnt model for *A. cochinchinensis* (Phillips et al., 2017). The MaxEnt model was constructed on the basis of data from 259 distribution sites and the top nine climate variables with their contributions. To ensure that the probability distribution of *A. cochinchinensis* was close to a normal distribution, we selected 70% of the data for model training and the remaining 30% for model validation. The maximum number of parameter repetitions was 10,000, with each process repeated 10 times and other parameters set at their default values (Wei et al., 2021).

In this study, the kuenm package in R was used to optimize the FC and RM in the MaxEnt model (Cobos et al., 2019). Firstly, the RM was set at intervals of 0.1 from 0.1 to 4.0 to generate 40 RM values. Then, four FCs, namely, linear feature (L), quadratic feature (Q), hinge feature (H), and product feature (P), were arranged and combined. Fifteen FC combinations (L, Q, H, P, LQ, LH, LP, QH, QP, HP, LQH, LQP, LHP, QHP, and LQHP) were thus obtained, and a total of 600 parameter combinations were obtained by combining the FC and RM values (Liu et al., 2019). To determine the best model, we selected statistically significant models where the omission rate was less than 0.05 and the delta_AICc (OR_AICc) value did not exceed 2 (Ye et al., 2018).

We also used the area under the receiver operating characteristic curve (i.e., the AUC) to assess the accuracy of model predictions (Lobo et al., 2007). The range of AUC values was (0,1), and when AUC > 0.9 the model results were regarded as very reliable. Moreover, we also considered the difference between the training AUC value and the testing AUC value. The smaller was the absolute value of this difference, the higher was the reliability of the model (Warren & Seifert, 2011).

### 2.3 Prediction of potentially suitable habitats, low‐impact areas, and changes in spatial patterns

A previous study (Tang et al., 2018) suggested that the MTSPS threshold is superior to other criteria for classifying suitable regions. The range of MTSPS values of suitable habitats for a species is usually (0,1), with higher values indicating that the species is more adapted to grow in a particular area. We used an MTSPS value as the threshold below which regions were defined as unsuitable. The areas above the threshold were divided into three groups corresponding to regions of low, moderate, and high suitability. The areas of suitable regions and the percentage changes in area between various eras were calculated.

A low‐impact area, also known as a relatively stable habitat, refers to a region where climate change will have less effect on species (Pan et al., 2020). In DIVA‐GIS v7.5.0, by overlaying maps of the predicted distributions of suitable habitats for the different eras (2041–2070 and 2071–2100) and climate scenarios (SSP1‐RCP2.6, SSP3‐RCP7, and SSP5‐RCP8.5) studied, we redefined regions where the probability of presence of the species was greater than the MTSPS threshold as suitable habitats for the species. We selected the overlapping parts of the overlay layer to visually display the potential range of relatively stable suitable habitats for *A. cochinchinensis*.

A change in spatial pattern refers to a change in the potential habitat of a species between different climate scenarios and eras, which is determined by overlaying maps of predicted habitats in different eras (Zurell et al., 2020). In this study, we used the SDMtoolbox v2.4 toolkit in ArcGIS v10.4 to analyze potential changes and calculate the changing trends in suitable habitats for *A. cochinchinensis* between current conditions and different SSP scenarios in different eras.

### 2.4 Centroid migration analysis

We used the SDMtoolbox v2.4 toolkit in ArcGIS v10.4 to calculate the changing trends of suitable habitats for *A. cochinchinensis* between different periods under different climate scenarios (Brown et al., 2017). In this study, the potentially suitable areas were simplified in the form of a vector center. Changes in the position of the centroid were used to indicate the directions of shifts in the suitable regions for *A. cochinchinensis*. The directions and distances of migrations of the suitable regions between different eras under different climate scenarios were also determined.

### 2.5 Chemical composition analysis

#### 2.5.1 Determination of the contents of the index compounds

The contents of two steroidal saponins (protodioscin and protogracellin) in the root tubers of *A. cochinchinensis* were determined by HPLC. About 1 g of dried *A. cochinchinensis* root tuber powder was added to 10 mL of 65% methanol and sonicated (300 W, 40 kHz) for 30 min, and the supernatant was filtered through a 0.22 μm filter membrane for HPLC analysis. Compounds were analyzed on an HPLC system (Shimadzu, Kyoto, Japan) equipped with a Hypersil BDS C18 column (Elite, Dalian, Liaoning, China) (250 × 4.6 mm, 5 μm). The mobile phase consisted of pure water (A) and acetonitrile (B) with a gradient elution concentration of 90% A (0 min), 90% A (7 min), 72% A (20 min), and 68% A (45 min). All separations were carried out at a constant column temperature of 30°C with a wavelength of 203 nm and a flow rate of 1.0 mL/min.

#### 2.5.2 Determination of the total polysaccharide content

The phenol–sulfuric acid method was used to determine the total polysaccharide content (Chu et al., 2024). In brief, 1 mL of an aqueous extract of *A. cochinchinensis* root tuber powder was placed in a test tube, and 1 mL of 5% phenol solution was then added. Next, 5 mL of concentrated sulfuric acid was quickly added, and the mixture was held in a water bath at 40°C for 30 min. After the mixture was taken out, the reaction was terminated in an ice‐water bath for 5 min, and the absorbance at 490 nm was determined. The total polysaccharide content (*x*) was calculated on the basis of the concentration of a glucose standard, as shown in Equation (1):

(1) $$x\%=\left(\frac{\mathrm{cVn}}{m}\right)\times 100\%$$

where *c* is the concentration of the glucose standard (mg/mL), *V* is the volume of the sample solution (mL), *m* is the mass of the root tuber sample (g), and *n* is the dilution factor.

#### 2.5.3 Determination of the total saponin content

The total saponin content was determined using the colorimetric vanillin–perchloric acid reaction (Tian et al., 2020). A 1 mL portion of a methanolic extract of a root tuber sample purified using a water‐saturated *n*‐butanol solution was taken, and the methanol was removed by drying. Then 0.2 mL of a 5% vanillin‐glacial acetic acid solution and 0.8 mL of perchloric acid were added, and the mixture was placed in a water bath at 60°C for 15 min and then immediately transferred to an ice bath for 5 min. Next, 5 mL glacial acetic acid was added to terminate the reaction, and then the mixture was allowed to stand for 10 min to measure the absorbance at 455 nm. On the basis of the concentration of a dioscin standard, the total content of saponins (*y*) was calculated as follows:

(2) $$y\%=\left(\frac{c^{\prime }V^{\prime }N}{M}\right)\times 100\%$$

where *c*' is the concentration of the dioscin standard (mg/mL), *V*′ is the volume of the sample solution (mL), *M* is the mass of the root tuber sample (g), and *N* is the dilution factor.

### 2.6 Analysis of correlations between the chemical composition and climatic factors

We used SPSS 22.0 to analyze the correlations between the contents of active ingredients in *A. cochinchinensis* and the abovementioned 47 climatic factors. These correlations were measured using the Spearman correlation coefficient and finally visualized using Cytoscape v3.8.0.

## 3 RESULTS

### 3.1 Analysis of contributions of the environmental variables and accuracy of the model

From the screening of 47 candidate climate variables based on their contribution rankings, we ultimately obtained nine climate variables for construction of the model, as shown in Table 1. The percentage contributions of these variables were as listed in Table 1, (Figures S1 and S2).

**TABLE 1:** Environmental variables used for model construction.

Variable	Percent contribution (%)	Note
npp	30.1	Net primary productivity
vrm	20.4	Vector ruggedness measure
PETColdestQuarter	17.8	Mean monthly potential evapotranspiration during the coldest quarter
PETWarmestQuarter	12.7	Mean monthly potential evapotranspiration during the warmest quarter
bio14	12.5	Amount of precipitation in the driest month
bio04	3.6	Temperature seasonality
bio05	1.6	Mean daily maximum air temperature in the warmest month
aridityIndexThornthwaite	0.7	Thornthwaite aridity index
annualPET	0.5	Annual potential evapotranspiration

After optimization using the kuenm package in R, the model predicted the best results when the feature class (FC) was PH and the regularization multiplier (RM) was 3.5 (Table 2). At this point, in the model the training area under the curve (AUC) value was 0.9350 ± 0.017, the testing AUC value was 0.9370 ± 0.0300, and the absolute difference between the training AUC value and the testing AUC value (|AUCDIFF|) was thus 0.0020, which demonstrated excellent prediction of results (Figure S3).

**TABLE 2:** The best model parameters after optimization.

Model	Feature class	Regularization multiplier	Omission_rate_at_5%	Delta_AICc
Default	LQPH	1	0.025641	58.991177
Best_model	PH	3.5	0.025641	0

### 3.2 Current potential suitable areas

On the basis of the maximum test sensitivity plus specificity (MTSPS) threshold (0.2743), we divided the space into four parts: MTSPS = 0–0.2743 indicated unsuitable regions; MTSPS = 0.2743–0.5162 corresponded to regions of low suitability; MTSPS = 0.5162–0.7581 corresponded to regions of moderate suitability; and MTSPS = 0.7581–1 indicated regions of high suitability.

**FIGURE 2:**
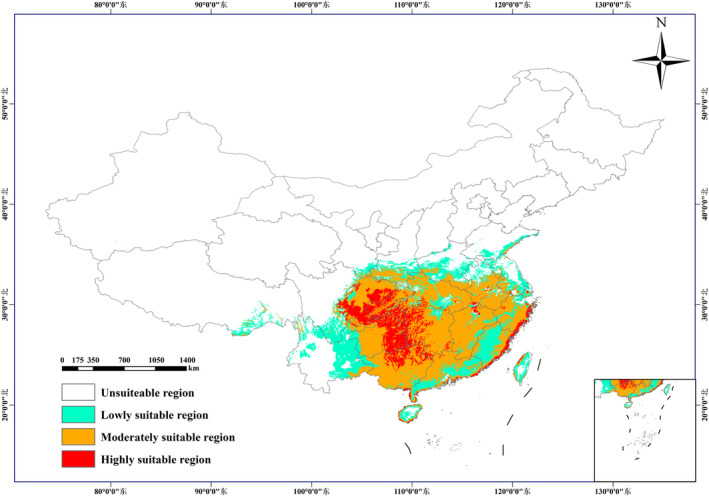
Prediction of the distribution pattern of suitable growing areas for *Asparagus cochinchinensis* under current climate conditions (1981–2010).

As shown in Figure 2, the area of the current potential suitable regions for *A. cochinchinensis* in China is 208.38 × 10^4^ km^2^. These are mainly located in southern provinces such as Sichuan, Yunnan, Guizhou, Guangxi, Guangdong, Chongqing, Hubei, Hunan, Jiangxi, Fujian, and Zhejiang. In addition, they are distributed in other areas such as Jiangsu, Shandong, Shaanxi, Henan, and Taiwan. The regions of high, moderate, and low suitability have areas of 30.57 × 10^4^ km^2^, 113.65 × 10^4^ km^2^, and 64.15 × 10^4^ km^2^, respectively. The areas of high suitability are mainly located in Sichuan, Chongqing, Guizhou, and Guangxi.

### 3.3 Future potential suitable areas

**FIGURE 3:**
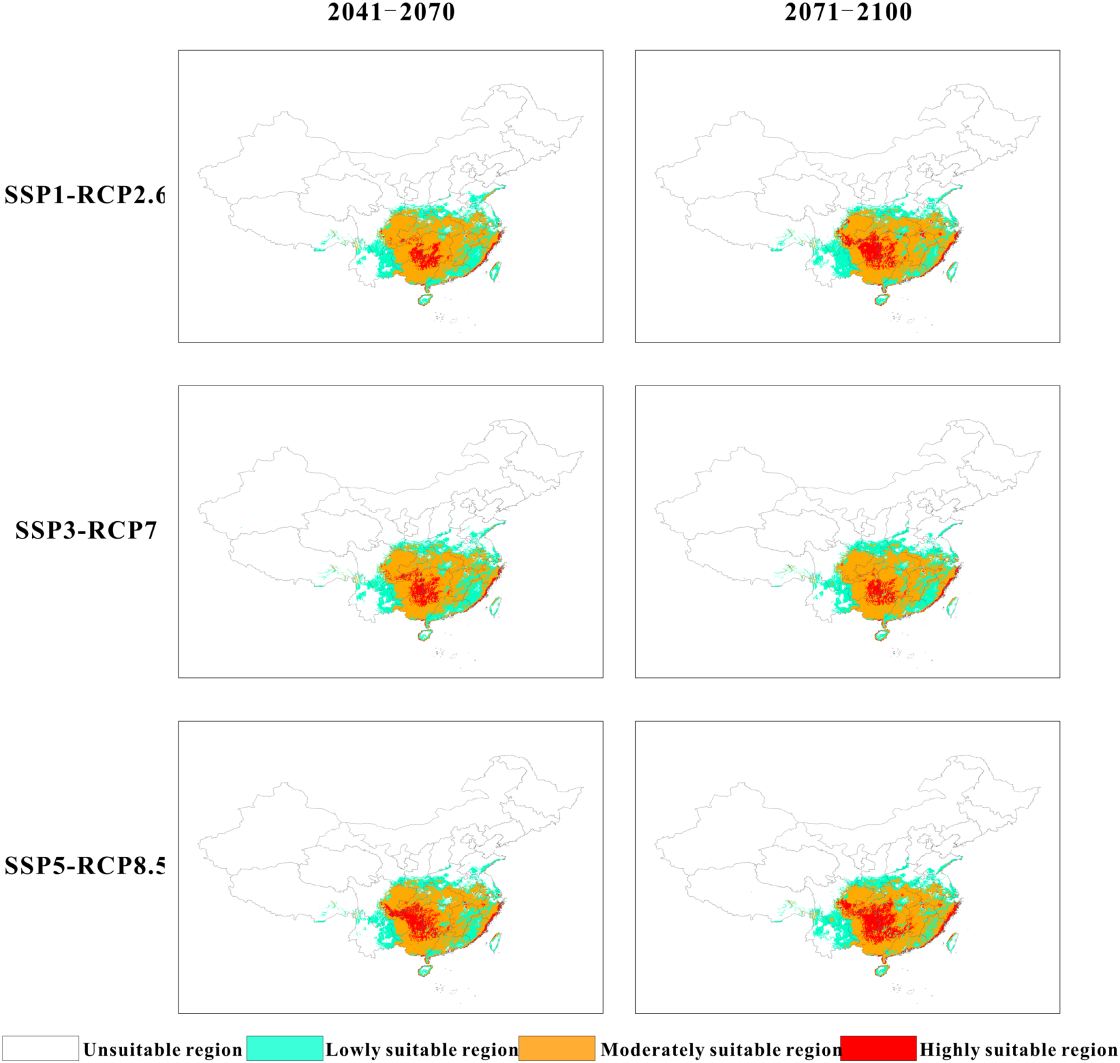
Prediction of the distribution of suitable areas for *Asparagus cochinchinensis* in future climate scenarios (2041–2100).

**FIGURE 4:**
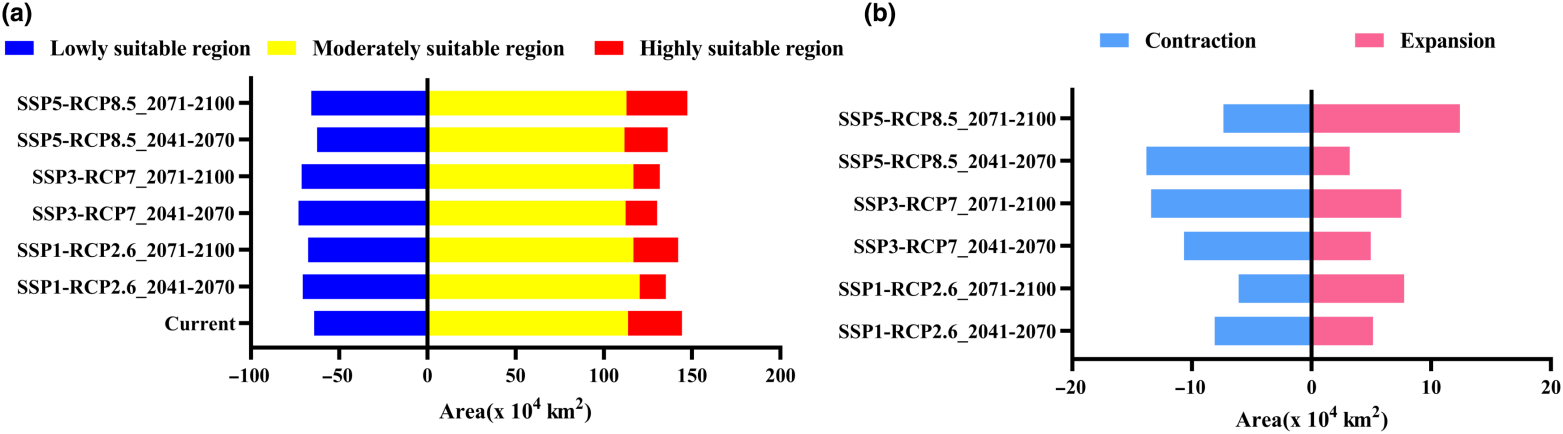
The areas (a) and changes (b) of suitable areas for *Asparagus cochinchinensis* in different future years and climate scenarios.

The potential distribution of *A. cochinchinensis* and changes in suitable habitats for *A. cochinchinensis* in the next two decades will vary depending on different climate scenarios (Figure 3, Figure S4). Under different climate scenarios, the area of highly suitable habitats will decrease considerably in comparison with the current value except for a slight increase in 2071–2100 under the shared socioeconomic pathway 5 (SSP5)‐representative concentration pathway 8.5 (RCP8.5) scenario, while the areas of regions of low and intermediate suitability will change little. As shown in Figure 4, under the SSP1‐RCP2.6 scenario, the total area of suitable habitats for *A. cochinchinensis* will remain almost unchanged (98.73%–100.65%), with small increases in the areas of regions of both low and moderate suitability. In contrast, the area of highly suitable regions will decrease significantly. In 2041–2070, the area of highly suitable regions will be only 14.79 × 10^4^ km^2^, which corresponds to only 48.38% of the current area of highly suitable regions. Under the SSP3‐RCP7 scenario, there will be a slight decrease in the total area of suitable habitats, a slight increase in the area of minimally suitable habitats, and a significant decrease in the area of highly suitable habitats. Under the SSP5‐RCP8.5 scenario, the trends of changes in the total area of suitable habitats for *A. cochinchinensis* and the area of minimally suitable habitats are consistent and exhibit a slight decrease followed by an increase. The area of moderately suitable habitats will undergo a slight decrease, while the area of highly suitable habitats will experience a significant decline, followed by an increase (79.80%–112.86%) (Table S3).

### 3.4 Identification of low‐impact areas

**FIGURE 5:**
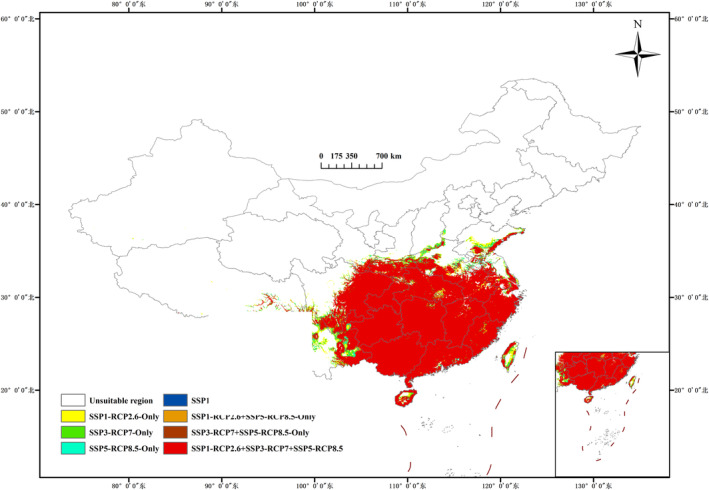
Comprehensive prediction of low‐impact areas in *Asparagus cochinchinensis* under different climate scenarios.

As the severity of climate change increases (SSP1‐RCP2.6 → SSP3‐RCP7 → SSP5‐RCP8.5), the total area of regions of low impact for *A. cochinchinensis* will remain almost unchanged (205.23 × 10^4^ km^2^ → 203.14 × 10^4^ km^2^ → 206.70 × 10^4^ km^2^). As shown in Figure 5, the results of predictions indicate that Chongqing, Guangxi, Guizhou, Hubei, Hunan, Jiangxi, Zhejiang, Fujian, Shaanxi, the southern part of Henan, the central and southern parts of Anhui, the coastal zones of Hainan and Taiwan, the northeastern part of Yunnan, and the Sichuan Basin will always be relatively stable suitable areas for the growth of *A. cochinchinensis* under any climate scenario. Under the scenario combination of SSP1‐RCP2.6 + SSP3‐RCP7 + SSP5‐RCP8.5, the area of low impact will be the smallest and will correspond to 92.07% of the current suitable area.

### 3.5 Migration of the centroid of the potentially suitable regions

**FIGURE 6:**
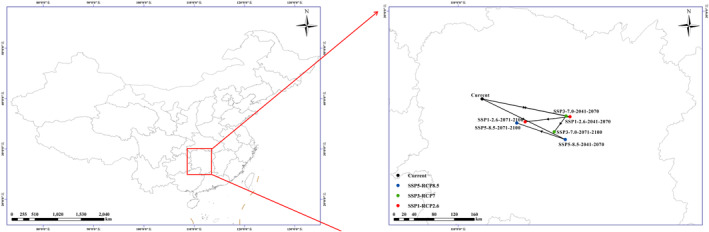
Migration of *Asparagus cochinchinensis* centroids under different climate scenarios.

From the overall results predicted by the model, it can be seen that under the SSP1‐RCP2.6 and SSP3‐RCP7 climate scenarios the migration of the centroid of the potentially suitable regions for *A. cochinchinensis* will be similar to, but different from, that under the SSP5‐RCP8.5 climate scenario (Figure 6). Under the SSP1‐RCP2.6 and SSP3‐RCP7 scenarios, the centroid of the potentially suitable areas will move first 174.88 and 166.89 km (2041–2070), respectively, to the southeast and then 89.25 and 23.40 km (2071–2100) to the southwest over time, respectively. However, under the SSP5‐RCP8.5 scenario, the centroid of the potentially suitable areas will move first to the southeast by 165.08 km (2041–2070) and then to the northwest by 96.33 km (2071–2100).

### 3.6 Accumulation patterns of major secondary metabolites in *Asparagus cochinchinensis* from different regions

**FIGURE 7:**
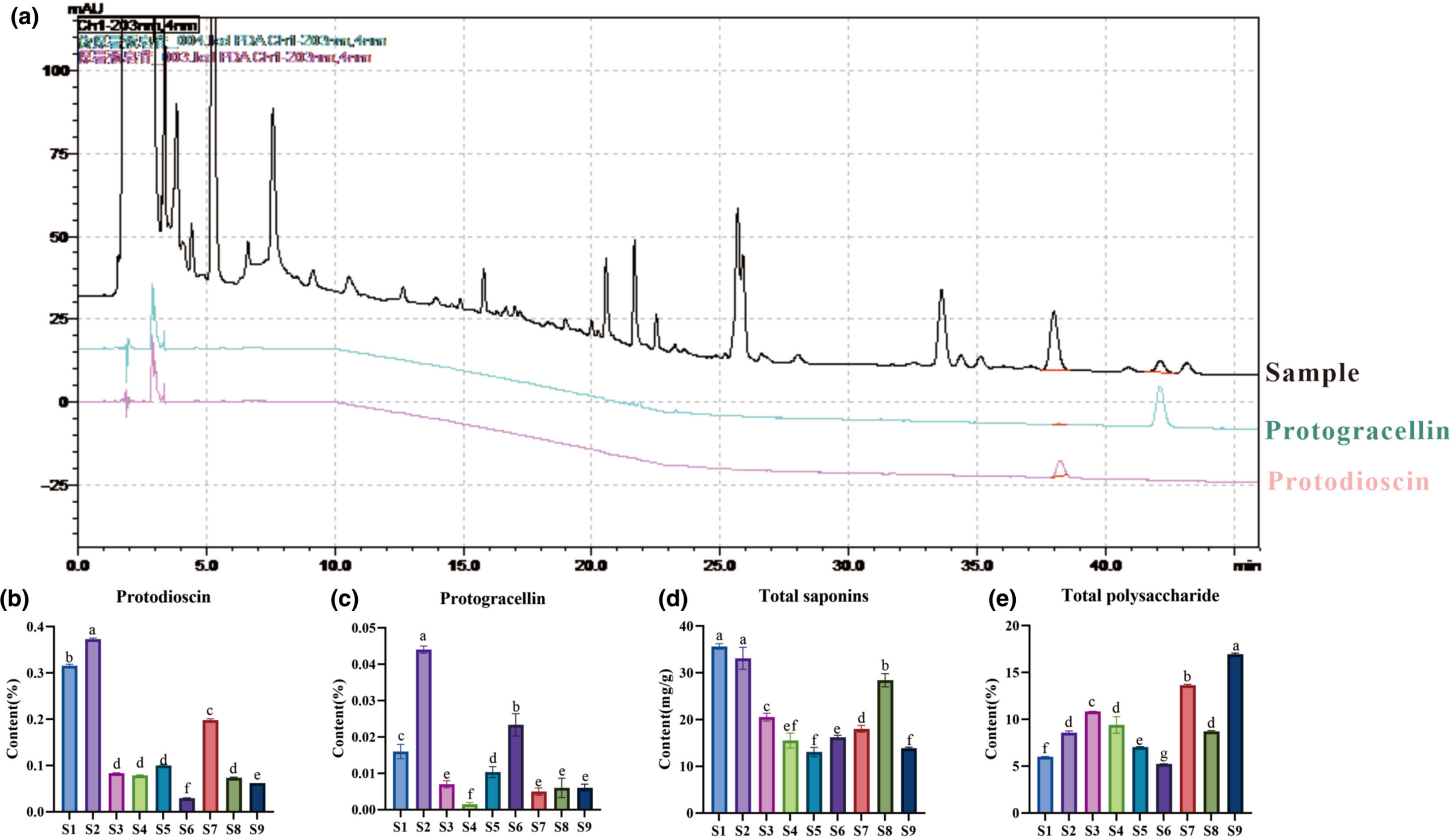
Determination of active ingredient content in *Asparagus cochinchinensis* tuber roots. (a) HPLC chromatograms of references and samples. (b–e) The contents of protodioscin (b), protogracillin (c), total saponins (d), and total polysaccharide (e).

The contents of secondary metabolites in samples of *A. cochinchinensis* root tubers from different cultivation areas varied (Figure 7). The changes in the contents of protodioscin and protogracellin were similar, with the contents in samples S1 and S2 being higher. One difference was that S7 had a higher protodioscin content, and S6 had a higher protogracellin content, whereas the contents of these two saponins in the remaining samples were low (Figure 7b,c). In terms of the total content of saponins, samples S1, S2, and S8 were found to have high contents, whereas the contents in samples S5 and S9 were low. However, the total contents of polysaccharides were significantly different, with higher contents in samples S9, S7, and S3 and lower contents in S1 and S6 (Figure 7d,e).

### 3.7 Correlations between metabolite accumulation and environmental factors

**FIGURE 8:**
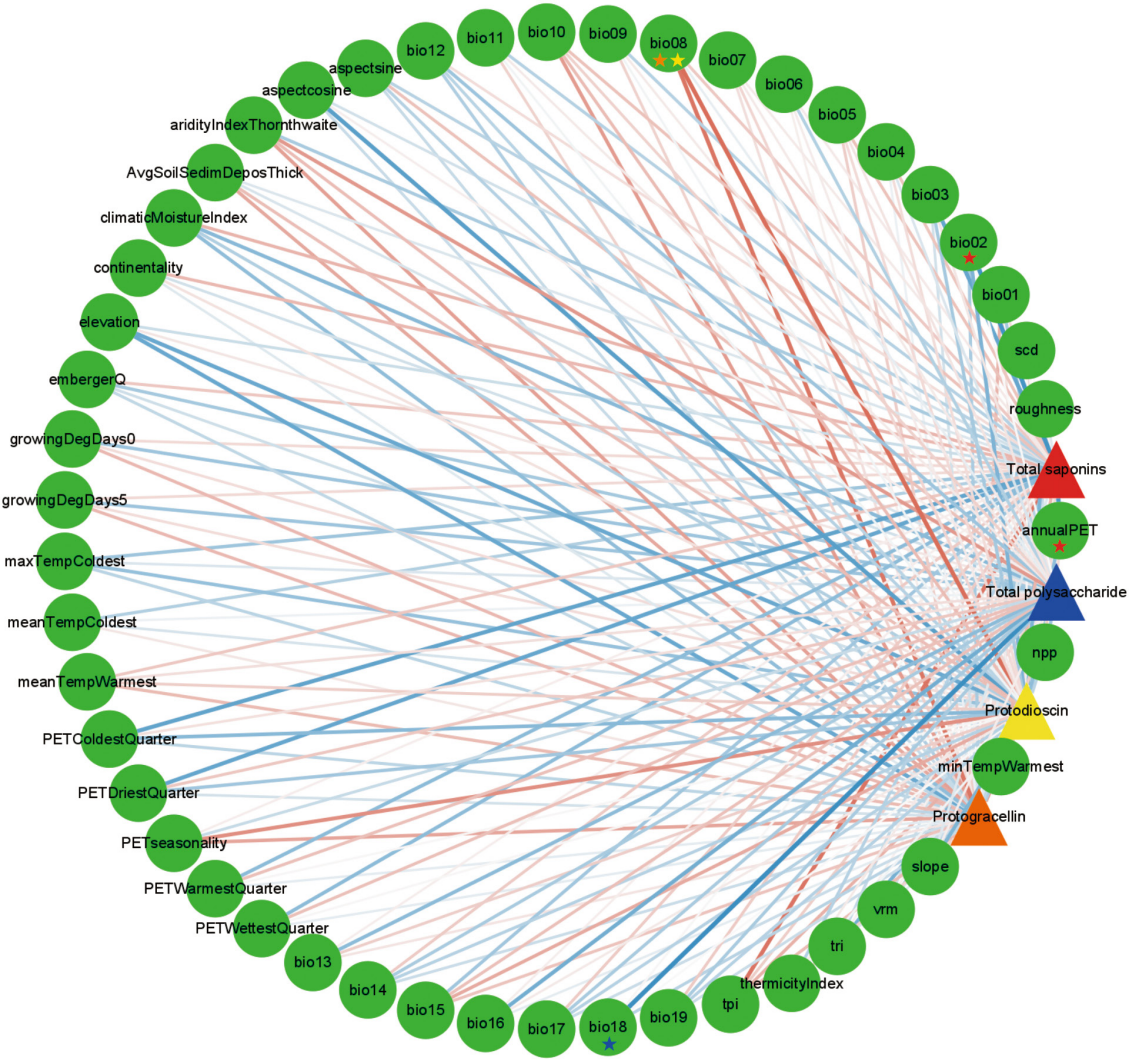
Correlation network between 47 environmental variables and the content of active components of *Asparagus cochinchinensis* based on the Spearman's correlation coefficient. The circles represent the environmental factors and the triangles represent the chemical components. Red indicates a positive correlation, blue indicates a negative correlation, and the line thickness indicates the strength of the correlation. ★ on the climate factor indicates significant difference from the chemical composition of the same color (*p* < .05).

The total contents of saponins and polysaccharides in the root tubers of *A. cochinchinensis* were strongly correlated with different environmental variables. The correlations between the contents of major secondary metabolites and 47 ecological variables are shown in Figure 8. The results demonstrate that the total saponin content was significantly negatively correlated with annualPET (annual potential evapotranspiration) and bio02 (mean daily air temperature range) (*p* < .05) and the total polysaccharide content was significantly negatively correlated with bio18 (mean monthly amount of precipitation during the warmest quarter) (*p* < .05). In addition, the concentrations of both protodioscin and protogracellin were positively associated with bio08 (mean daily air temperature during the wettest quarter) (*p* < .05).

## 4 DISCUSSION

### 4.1 Rationality and accuracy of MaxEnt model predictions

The MaxEnt model has become one of the most widely used models of species distribution because of the stability of its simulation results and its high accuracy (Phillips et al., 2006). This model calculates the relationships between the spatial distribution of species and environmental variables to predict the impacts of climate change on the distribution of suitable plant habitats (Estes et al., 2013).The reason why only the existing latitude and longitude data for species distribution sites were used as input variables was to avoid overfitting, even with incomplete data or small sample sizes, and to maintain stable and reliable accuracy of model predictions (Abolmaali et al., 2018). When RM = 3.5 and FC = PH, the delta_AICc value of the model was lower than the value with the default parameters (RM = 1 and FC = LQHPT), which indicated that the complexity and the extent of overfitting of our optimized model were low. The average AUC of the data from 10 simulations using the model was 0.936, and the standard deviation was 0.012, which demonstrated that the model exhibited high accuracy. Therefore, our optimized predictive model was reliable and accurate.

### 4.2 Areas suitable for current and future cultivation of *Asparagus cochinchinensis*

According to the MaxEnt model, the core suitable habitats (highly suitable habitats) for *A. cochinchinensis* are mainly located in the southeast of the Sichuan Basin, the southwest of Chongqing, the east of Guizhou, the north of Guangxi, and the southern coastal areas, which is consistent with previous studies (Liang et al., 2018; Lu & Liu, 2020; Xue et al., 2022). Precipitation, temperature, and soil conditions are the main factors affecting the distribution of medicinal plants (Feng et al., 2023). However, there are variations in the main factors determining the potential suitability of habitats for different medicinal plants. For example, extreme temperatures and precipitation were the most critical factors affecting the probable distribution of *S. baicalensis* (Xu et al., 2020). This was slightly different from the findings of our research. We found that the growth and distribution of *A. cochinchinensis* are mainly influenced by factors such as the carbon sequestration capacity (in terms of npp), terrain roughness (i.e., vrm), and atmospheric evapotranspiration (PETColdestQuarter, PETWarmestQuarter, and annualPET), as well as temperature and precipitation (bio14, bio04, and bio05).

In this study, we found that the most critical factor affecting the distribution of *A. cochinchinensis* is npp (the net primary productivity of plants), which represents the amount of new carbon fixed by photosynthesis per unit space and per unit time in a plant community (Sun et al., 2022). Variations in npp are mainly limited by light exposure, altitude, temperature, and precipitation (Zhou et al., 2020). Field investigations and related studies found that *A. cochinchinensis* preferentially grows in warm and humid areas without continuous exposure to light. Specifically, an average annual temperature of 16–18°C, an average annual precipitation of 960 mm, and an average annual humidity of 80% comprise favorable conditions for the growth of *A. cochinchinensis* (Yang, Zhang, et al., 2023). This is consistent with the results predicted by the MaxEnt model in this study and further confirms the accuracy of the model.

In the coming decades, the total area of suitable habitats, area of minimally suitable habitats, and area of moderately suitable habitats for *A. cochinchinensis* will not change greatly. In contrast, the area of the core suitable habitats (highly suitable habitats) will change significantly. Under the SSP1‐RCP2.6 and SSP3‐RCP7 climate scenarios, the area of core suitable habitats for *A. cochinchinensis* will decrease considerably, and the centroid of the suitable regions will move first to the southeast and then to the southwest. However, under the SSP5‐RCP8.5 scenario the area of core suitable habitats for *A. cochinchinensis* will decrease slightly and then increase to 1.13 times the current value. The centroid will migrate initially to the southeast and then to the northwest. The above results suggest that under a future climate scenario of high carbon emissions, *A. cochinchinensis* would eventually migrate and adapt to the high‐altitude, arid northwestern region. This is similar to the results for *Gastrodia elata* Blume, where the area of suitable habitats will increase under the SSP5‐RCP8.5 climate scenario (Guo et al., 2022). Similarly, it was predicted that suitable areas for *Paeonia lactiflora* Pall. under the SSP5‐RCP8.5 scenario would eventually extend to high altitudes and latitudes, which is similar to our results (Zhang et al., 2019).

### 4.3 Effects of environmental factors on the quality of medicinal plants

It is well known that environmental factors play essential roles in the accumulation of secondary metabolites in medicinal plants (Appiah et al., 2023; Ncube et al., 2012; Zhang et al., 2020). The correlations between the nine *A. cochinchinensis* samples in this study and different climatic factors confirmed this opinion. Among these correlations, there was a significant negative correlation between the total polysaccharide content and the precipitation during the hottest quarter. Specifically, at higher temperatures, the smaller was the amount of precipitation, the greater was the total content of polysaccharides that accumulated in *A. cochinchinensis*. In contrast, as the amount of precipitation increased, the total polysaccharide content decreased. In addition, there was a significant negative correlation between the total saponin content and the mean daily temperature range, which indicated that an increase in extreme temperatures would reduce the accumulation of saponins in *A. cochinchinensis*. Regarding the index compounds in *A. cochinchinensis*, the contents of both protodioscin and protogracellin were significantly positively correlated with the mean temperature during the wettest quarter. This indicated that in situations with higher precipitation and humidity, the higher was the temperature, the greater were the amounts of these two compounds that accumulated. This was in contrast to the results for the total polysaccharide content, which demonstrated that the contents of these saponins and the total polysaccharide contents exhibited different patterns of response to the effects of climatic factors, which has not been found in previous studies. The above results confirmed that a warm and humid environment promoted the accumulation of active ingredients in *A. cochinchinensis*. Some studies have found that medicinal plants in highly suitable areas had higher contents of active components (Li et al., 2020; Shen et al., 2021; Yang, He, et al., 2023). This was consistent with our study and indicated that climatic conditions in highly suitable regions had a positive effect on the accumulation of active components in medicinal plants. However, some researchers have presented contrasting results, as in the case of *Panax notoginseng* (Zhan et al., 2022), which would have been caused by an increase in biomass in the medicinal parts of *P. notoginseng* in the highly suitable areas.

The strengths of this study are primarily attributed to the innovative integration of the MaxEnt model with HPLC analysis, which enhances the predictive power of species distribution models in identifying suitable areas, offering a more compelling approach than reliance on the model alone. Additionally, we performed a correlation analysis between the chemical composition of *A. cochinchinensis*, including its polysaccharides and saponins, and climate variables, pinpointing the climatic factors influencing these components—a discovery absent in previous research. Nevertheless, the study is not without its limitations. Notably, the chemical analysis was confined to medicinal active ingredients such as polysaccharides and saponins. Given that *A. cochinchinensis* has been recognized as a medicinal and edible variety by the National Health Commission of China, it would be beneficial to broaden the scope to include food evaluation indices like amino acids and total proteins for a more holistic assessment of the quality. Despite these considerations, the implications of our findings are substantial. This research elucidates the future distribution of suitable habitats for *A. cochinchinensis* under changing climatic conditions and identifies the climatic determinants of active component accumulation, thereby providing guidance for situating the core production areas of its artificial cultivation.

## 5 CONCLUSIONS

A MaxEnt model was established and optimized, and the distribution and future migration of potential suitable habitats for *A. cochinchinensis* in China were predicted. The total area of suitable regions for *A. cochinchinensis* will remain almost constant from the present to different future eras under different climate scenarios. The area of highly suitable habitats would eventually increase under the high‐carbon‐emissions SSP5‐RCP8.5 climate scenario. The southeastern Sichuan Basin, northern Guangxi, eastern Guizhou, and western Hunan will be highly suitable areas regardless of the climate scenario, and could therefore be considered the core areas for cultivation of *A. cochinchinensis*. The contents of secondary metabolites in *A. cochinchinensis* were associated with climatic factors. The total contents of saponins and polysaccharides were significantly, but oppositely, correlated with temperature, precipitation, and other factors. This study could provide guidance for resource protection planning, large‐scale cultivation, and improvements in the quality of *A. cochinchinensis*.

## AUTHOR CONTRIBUTIONS

**Tong Zhang:** Conceptualization (lead); data curation (lead); methodology (equal); writing – original draft (lead); writing – review and editing (lead). **Xiangyang Lv:** Resources (lead). **Qian Zhao:** Data curation (equal); methodology (equal). **Caijuan Zhang:** Methodology (equal); writing – original draft (equal); writing – review and editing (equal). **Honglin Yin:** Methodology (equal). **Shuyu Deng:** Data curation (equal); methodology (equal). **Gui Yan:** Data curation (equal). **Guangzhi Wang:** Investigation (supporting); methodology (equal). **Xiaoyan Cao:** Investigation (supporting). **Hong Ou:** Data curation (supporting); methodology (supporting); writing – review and editing (supporting). **Gang Shen:** Conceptualization (supporting); data curation (equal); methodology (equal); writing – original draft (equal); writing – review and editing (equal).

## CONFLICT OF INTEREST STATEMENT

The authors declare that there are no conflicts of interest.

## Supporting information


Data S1.

## Data Availability

All data for this study (including all environmental variables, samples collection information, model optimization parameters and the changes of suitable areas) can be found in the manuscript and Supporting Information (Data S1); raw data supporting the results of this study are available upon reasonable request.
