# Supplementary material for: Assessment of suitable region of Asparagus cochinchinensis (Lour.) Merr. under different climatic conditions in China by the MaxEnt model and HPLC analysis
Source: Ecol Evol. 2024 Oct 3;14(10):e70354. doi: 10.1002/ece3.70354 (PMC11449629; doi:10.1002/ece3.70354)
Supplement: Supplementary file 1 — Data S1. [file ECE3-14-e70354-s001.docx]

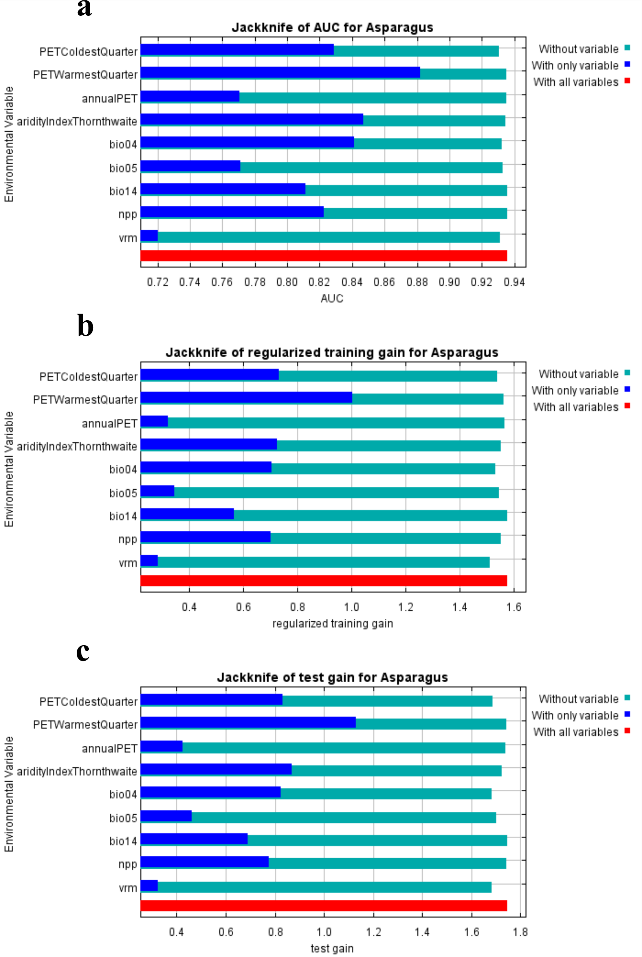


**FIGURE S1** Jackknife test of the importance of variables. Blue, green, and red bars represent running the MaxEnt model with the variable alone, without the variable, and with all variables, respectively. (a): AUC; (b): regularization training gain; (c): Test gain.


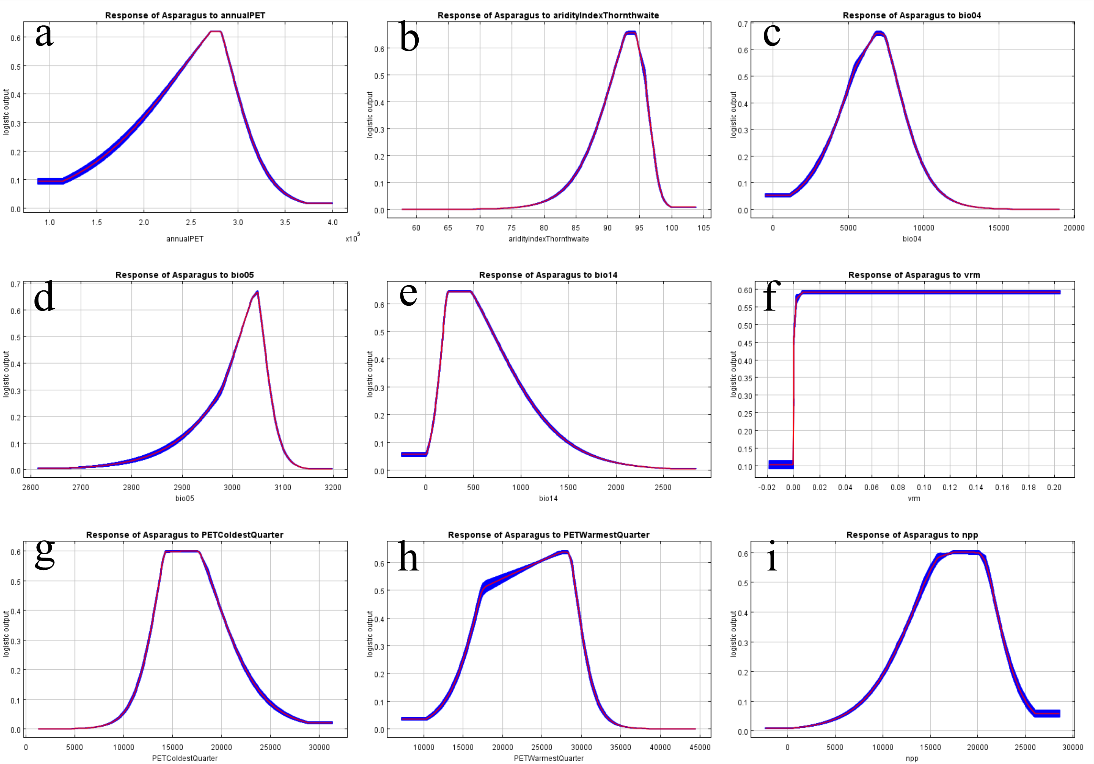


**FIGURE S2** Response curves of nine environmental predictors used in the ecological niche model for *A. cochinchinensis*. (a): annuelPET; (b): aridityIndexThornthwaite; (c): bio04; (d): bio05; (e): bio14; (f): vrm; (g): PETColdestQuarter; (h): PETWarmestQuarter; (i): npp.


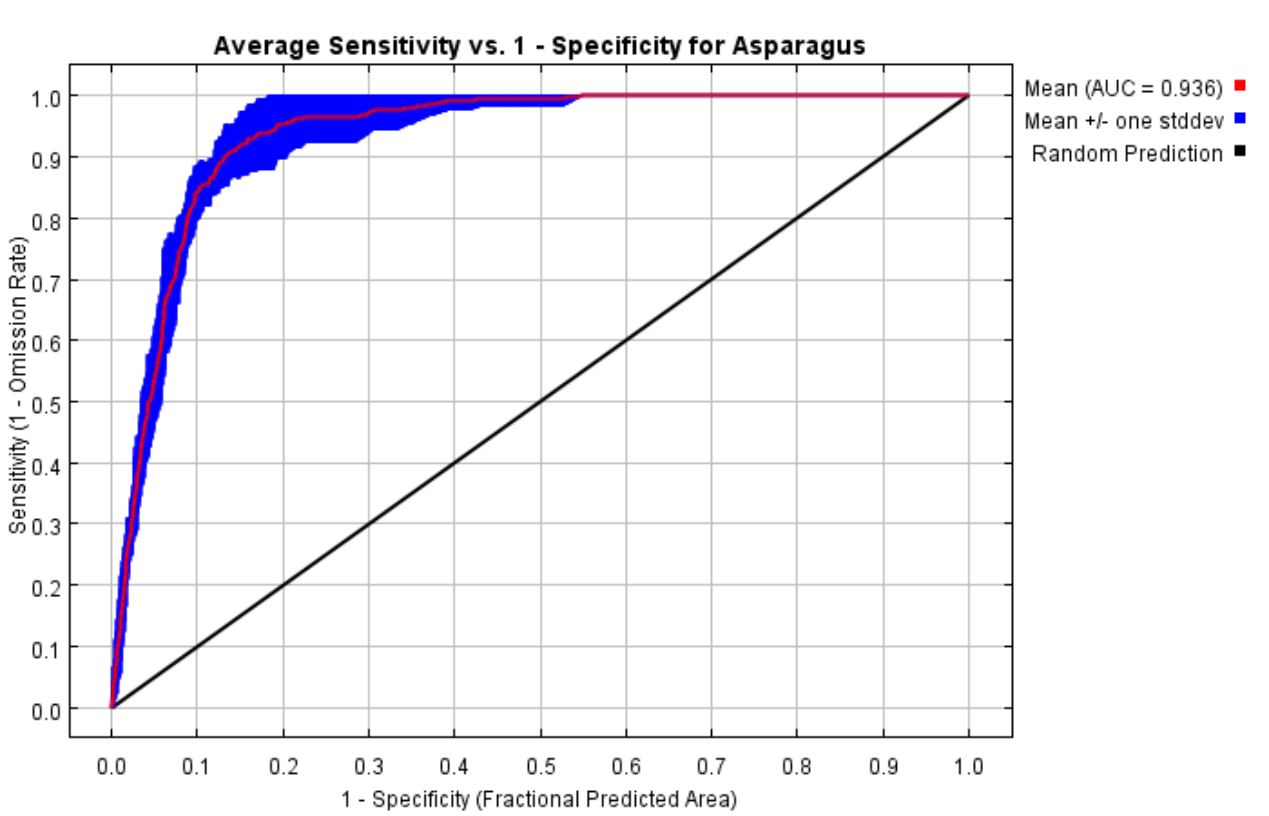


**FIGURE S3** Prediction validation with receiver operator characteristic (ROC) curves using the
MaxEnt model. AUC: the area under curve.


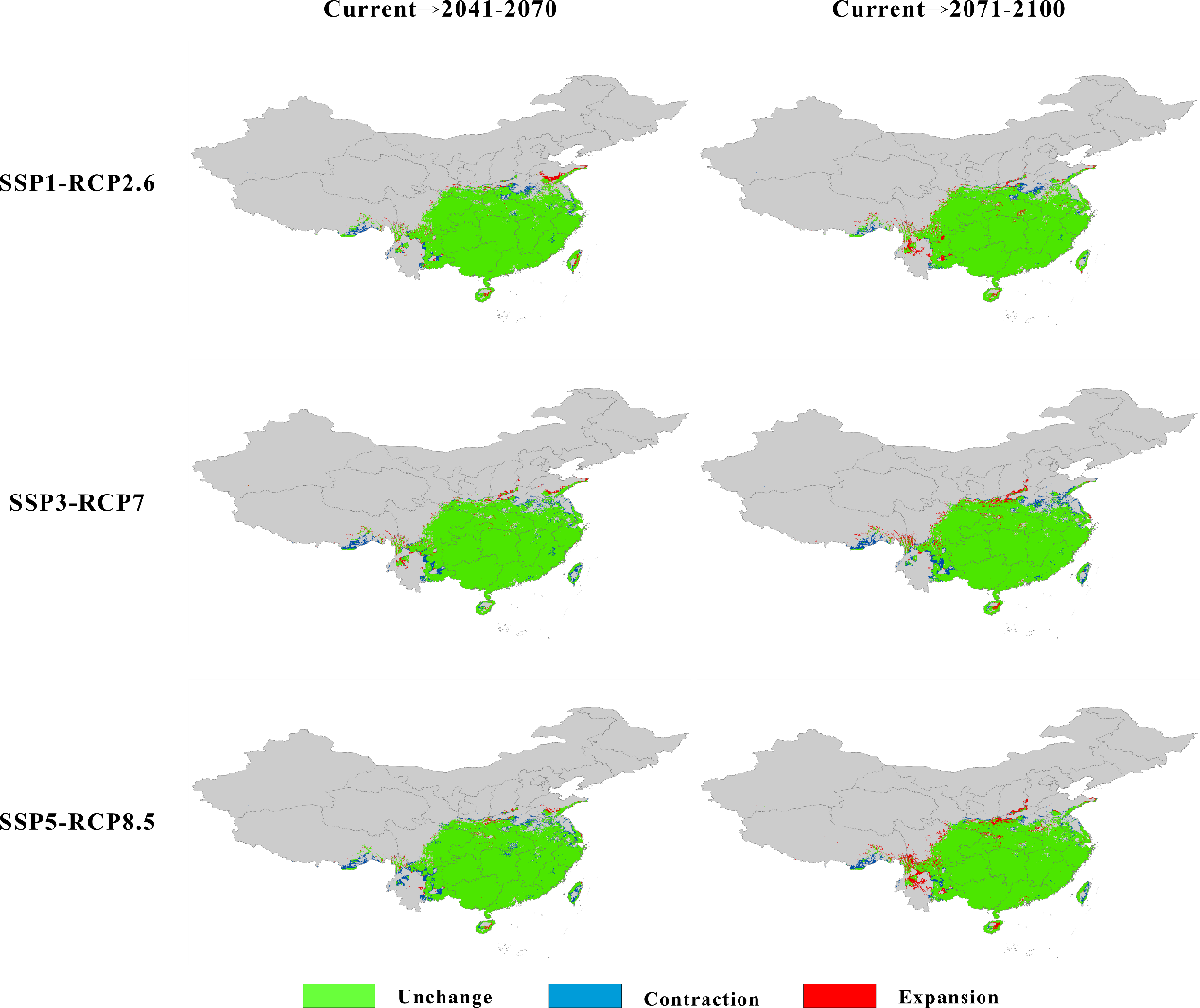


**FIGURE S4** Changes of potential suitable areas of *A. cochinchinensis* from current to future climatic conditions.

**TABLE S1** Samples collection location and latitude and longitude information.

| **No.** | **Region** | **Longitude/E** | **Latitude/N** |
| --- | --- | --- | --- |
| S1 | Dongxing District, Neijiang City, Sichuan Province | 105°4'28.524" | 29°35'36.456" |
| S2 | Zizhong County, Neijiang City, Sichuan Province | 104°51'7.632" | 29°45'50.976" |
| S3 | Shizhong District, Neijiang City, Sichuan Province | 105°4'3.936" | 29°35'13.128" |
| S4 | Longchang, Neijiang City, Sichuan Province | 105°17'15.828" | 29°20'22.128" |
| S5 | Pingshan County, Yibin City, Sichuan Province | 104°9'4.500" | 28°39'11.232" |
| S6 | Fusian District, Yulin City, Guangxi Province | 110°3'4.500" | 22°34'46.452" |
| S7 | Xingbin District, Laibin City, Guangxi Province | 109°14'4.632" | 23°43'38.712" |
| S8 | Donggua town, Chuxiong Yi Autonomous Prefecture, Yunnan Province | 101°31'39.612" | 25°2'41.820" |
| S9 | Pengshui Miao Tujia Autonomous County, Chongqing | 108°9'55.980" | 29°17'37.536" |

**TABLE S2** Information on 47 climate variables.

| **No.** | **Data type** | **Abbreviation** | **Full name** | **Unit** | **Explanation** |
| --- | --- | --- | --- | --- | --- |
| 1 | Continuous | bio01 | Mean annual air temperature | °C/10 | mean annual daily mean air temperatures averaged over 1 year |
| 2 | Continuous | bio02 | Mean diurnal air temperature range | °C/10 | mean diurnal range of temperatures averaged over 1 year |
| 3 | Continuous | bio03 | Isothermality | °C/10 | ratio of diurnal variation to annual variation in temperatures |
| 4 | Continuous | bio04 | Temperature seasonality | °C/10 | standard deviation of the monthly mean temperatures |
| 5 | Continuous | bio05 | Mean daily maximum air temperature of the warmest month | °C/10 | The highest temperature of any monthly daily mean maximum temperature |
| 6 | Continuous | bio06 | Mean daily minimum air temperature of the coldest month | °C/10 | The lowest temperature of any monthly daily mean maximum temperature |
| 7 | Continuous | bio07 | Annual range of air temperature | °C/10 | The difference between the Maximum Temperature of Warmest month and the Minimum Temperature of Coldest month |
| 8 | Continuous | bio08 | Mean daily mean air temperatures of the wettest quarter | °C/10 | The wettest quarter of the year is determined (to the nearest month) |
| 9 | Continuous | bio09 | Mean daily mean air temperatures of the driest quarter | °C/10 | The driest quarter of the year is determined (to the nearest month) |
| 10 | Continuous | bio10 | Mean daily mean air temperatures of the warmest quarter | °C/10 | The warmest quarter of the year is determined (to the nearest month) |
| 11 | Continuous | bio11 | Mean daily mean air temperatures of the coldest quarter | °C/10 | The coldest quarter of the year is determined (to the nearest month) |
| 12 | Continuous | bio12 | Annual precipitation amount | kg m^-2^ | Accumulated precipitation amount over 1 year |
| 13 | Continuous | bio13 | Precipitation amount of the wettest month | kg m^-2^ | The precipitation of the wettest month. |
| 14 | Continuous | bio14 | Precipitation amount of the driest month | kg m^-2^ | The precipitation of the driest month. |
| 15 | Continuous | bio15 | Precipitation seasonality | kg m^-2^ | The Coefficient of Variation is the standard deviation of the monthly precipitation estimates expressed as a percentage of the mean of those estimates (i.e. the annual mean) |
| 16 | Continuous | bio16 | Mean monthly precipitation amount of the wettest quarter | kg m^-2^ | The wettest quarter of the year is determined (to the nearest month) |
| 17 | Continuous | bio17 | Mean monthly precipitation amount of the driest quarter | kg m^-2^ | The driest quarter of the year is determined (to the nearest month) |
| 18 | Continuous | bio18 | Mean monthly precipitation amount of the warmest quarter | kg m^-2^ | The warmest quarter of the year is determined (to the nearest month) |
| 19 | Continuous | bio19 | Mean monthly precipitation amount of the coldest quarter | kg m^-2^ | The coldest quarter of the year is determined (to the nearest month) |
| 20 | Continuous | npp | Net primary productivity | g C m^-2^ y^-1^ 10^-1^ | Calculated based on the ‘Miami model’ [Lieth, H., 1972. "Modelling the primary productivity of the earth. Nature and resources", UNESCO, VIII, 2:5-10.] |
| 21 | Continuous | scd | Snow cover days | count | Number of days with snowcover calculated using the snowpack model implementation in from TREELIM (https://doi.org/10.1007/s00035-014-0124-0) |
| 22 | Continuous | annualPET | Annual potential evapotranspiration | mm / year | a measure of the ability of the atmosphere to remove water through evapotranspiration processes, given unlimited moisture |
| 23 | Continuous | aridityIndexThornthwaite | Thornthwaite aridity index | — | Index of the degree of water deficit below water need |
| 24 | Continuous | climaticMoistureIndex | climaticMoistureIndex | — | a metric of relative wetness and aridity |
| 25 | Continuous | continentality | continentality | °C | average temp. of warmest month - average temp. of coldest month |
| 26 | Continuous | embergerQ | Emberger's pluviothermic quotient | — | a metric that was designed to differentiate among Mediterranean type climates |
| 27 | Continuous | growingDegDays0 | growingDegDays0 | number of days | sum of mean monthly temperature for months with mean temperature greater than 0℃ multiplied by number of days |
| 28 | Continuous | growingDegDays5 | growingDegDays5 | number of days | sum of mean monthly temperature for months with mean temperature greater than 5℃ multiplied by number of days |
| 29 | Continuous | maxTempColdest | maxTempColdestMonth | ℃ * 10 | max. temp. of the coldest month |
| 30 | Continuous | meanTempColdest | Mean temp. of the coldest month | ℃ * 10 | mean temp. of the coldest month |
| 31 | Continuous | meanTempWarmest | Mean. temp. of the warmest month | ℃ * 10 | mean. temp. of the warmest month |
| 32 | Continuous | minTempWarmest | Min. temp. of the warmest month | ℃ * 10 | min. temp. of the warmest month |
| 33 | Continuous | PETColdestQuarter | Mean monthly PET of coldest quarter | mm / month | mean monthly PET of coldest quarter |
| 34 | Continuous | PETDriestQuarter | Mean monthly PET of driest quarter | mm / month | mean monthly PET of driest quarter |
| 35 | Continuous | PETseasonality | Monthly variability in potential evapotranspiration | mm / month | monthly variability in potential evapotranspiration |
| 36 | Continuous | PETWarmestQuarter | Mean monthly PET of warmest quarter | mm / month | mean monthly PET of warmest quarter |
| 37 | Continuous | PETWettestQuarter | Mean monthly PET of wettest quarter | mm / month | mean monthly PET of wettest quarter |
| 38 | Continuous | thermicityIndex | compensated thermicity index | ℃ | sum of mean annual temp., min. temp. of coldest month, max. temp. of the coldest month, x 10, with compensations for better comparability across the globe |
| 39 | Continuous | aspectcosine | Aspect Cosine | — | topography |
| 40 | Continuous | aspectsine | Aspect Sine | — |  |
| 41 | Continuous | elevation | Elevation | m |  |
| 42 | Continuous | roughness | Roughness | — |  |
| 43 | Continuous | slope | Slope | ° |  |
| 44 | Continuous | tpi | Topographic Position Index | — |  |
| 45 | Continuous | tri | Terrain Ruggedness Index | — |  |
| 46 | Continuous | vrm | Vector Ruggedness Measure | — |  |
| 47 | Continuous | AvgSoilSedimDeposThick | average_soil_and_sedimentary-deposit_thickness | m | Averages soil and sedimentary deposit thicknesses across upland hillslopes and valley bottoms in meters |

**TABLE S3** Changes in the suitable areas of *A. cochinchinensis* under future climate scenarios (2041-2100).

| Decades / SSPs | | Predicted area (× 10^4^ km^2^) and % of the corresponding current area | | | | | | | |
| --- | --- | --- | --- | --- | --- | --- | --- | --- | --- |
|  |  | Total suitable region | | Lowly suitable region | | Moderately suitable region | | Highly suitable region | |
| 1981-2010 | | 208.37 | - | 64.15 | - | 113.66 | - | 30.57 | - |
| SSP1-RCP2.6 | 2041-2070 | 205.74 | 98.73% | 70.67 | 110.15% | 120.28 | 105.83% | 14.79 | 48.38% |
|  | 2071-2100 | 209.74 | 100.65% | 67.60 | 105.37% | 116.70 | 102.68% | 25.44 | 83.22% |
| SSP3-RCP7 | 2041-2070 | 203.17 | 97.50% | 73.00 | 113.79% | 112.26 | 98.77% | 17.91 | 58.60% |
|  | 2071-2100 | 202.99 | 97.41% | 71.25 | 111.06% | 116.78 | 102.75% | 14.96 | 48.94% |
| SSP5-RCP8.5 | 2041-2070 | 198.58 | 95.30% | 62.48 | 97.39% | 111.71 | 98.29% | 24.39 | 79.80% |
|  | 2071-2100 | 213.08 | 102.26% | 65.80 | 102.57% | 112.78 | 99.23% | 34.50 | 112.86% |
